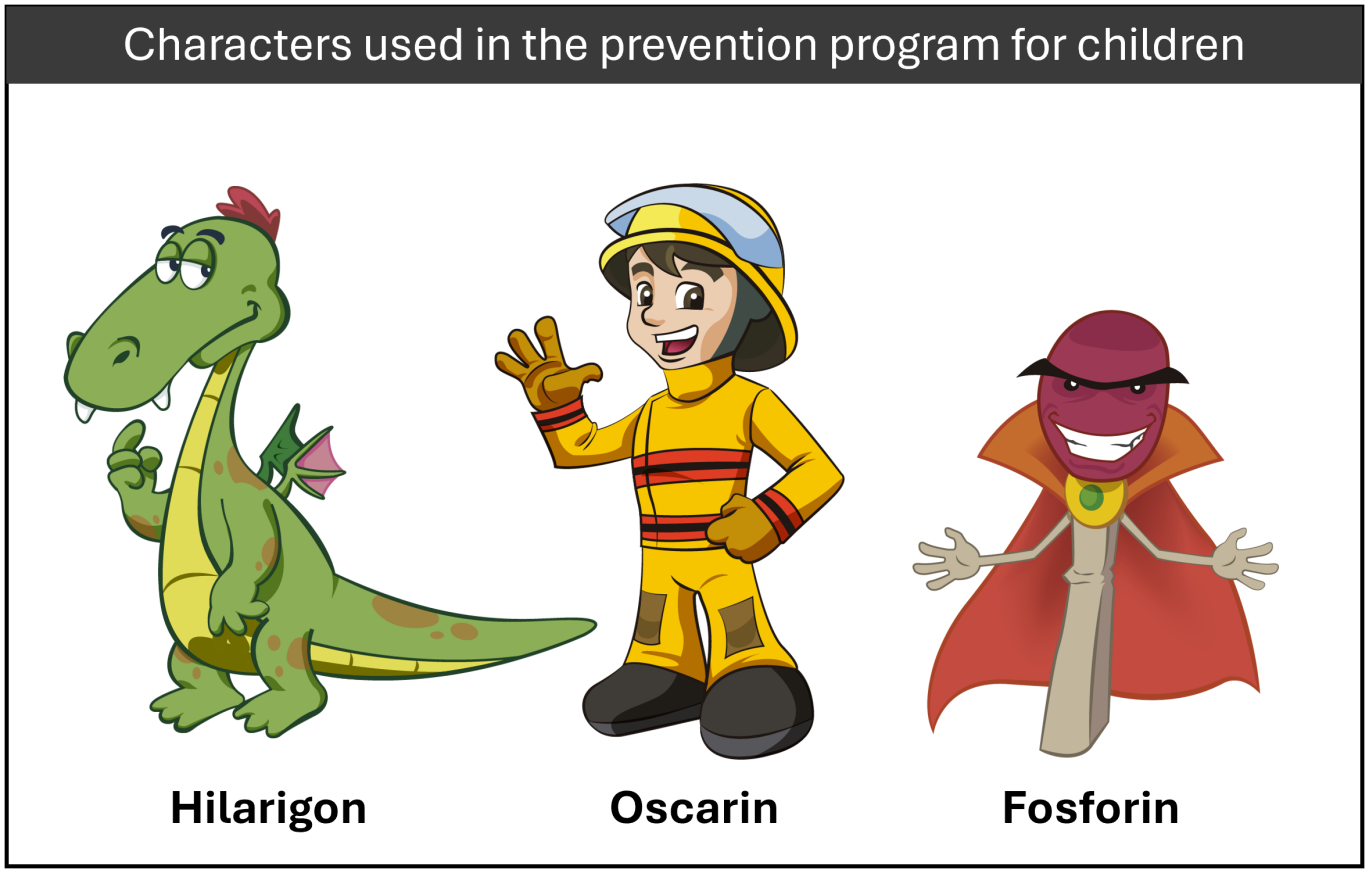# 122 Efficacy of a Burn Prevention Program to Decrease the Incidence of Burn Injuries in Children

**DOI:** 10.1093/jbcr/iraf019.122

**Published:** 2025-04-01

**Authors:** Hilarion Castañeda, Oscar Fabian Gutierrez-Tenorio, Rocio Muñoz-Sandoval, Elizabeth Lopez, Juan Manuel Marquez-Romero

**Affiliations:** Hospital MAC - Star Medica Aguascalientes; Grupo Solfis; Hospital MAC - Universidad Cuauhtemoc; Hospital General de Zona 1, IMSS; Hospital General de Zona 2, IMSS

## Abstract

**Introduction:**

Pediatric burn injuries pose a significant burden of disease, leading to substantial morbidity and mortality. This study aimed to evaluate the efficacy of the “No More Burns” educational program in preventing burn injuries among children under nine years old.

**Methods:**

The “No More Burns” program started in 2014 to teach long-term prevention behaviors and reduce burn accidents and deaths. The program has three pillars: educational courses for children, training for first-contact health workers, and specialized care for patients with burn sequelae. The epidemiological data for burn injuries in our state from 2014 to 2022 was obtained from the National System of Epidemiological Surveillance of the Health Ministry. We analyzed the monthly incidence rates per 100,000 inhabitants using the Mann-Kendall trend test and segmentation analysis to identify trends and changes.

**Results:**

The program trained over 25,000 scholars from 1750 elementary schools on burn prevention behaviors through animated short films, booklets, and comics. All the schools received a kit with medical supplies for immediate burn care. More than 2000 healthcare personnel and students were trained in caring for burn patients. Additionally, 200 children with burn sequelae were identified and scheduled to receive medical or surgical treatment and rehabilitation.

The analysis showed a significant decrease in the state incidence rate of burn injuries compared to the national mean post-implementation. The estimated number of burn injuries prevented during the study period was approximately 3,839, or 604 per year.

**Conclusions:**

The “No More Burns” program has significantly impacted burn prevention among children in our state. Despite the limitations of relying on government-reported data, the correlation between the program’s activities and the observed decrease in burn injuries is promising. These findings underscore the importance of the “No More Burns” program’s activities in achieving sustained, targeted educational interventions and long-term reductions in burn injury incidence. They also highlight the need for more robust study designs to validate these outcomes, emphasizing the importance of evidence-based strategies in future burn prevention efforts.

**Applicability of Research to Practice:**

Educational programs can increase awareness, reduce burn injuries, and promote better care, constituting a viable option to decrease the incidence of burn injuries. The “No More Burns” educational program was feasible and effective and can be replicated and adapted in other settings.

**Funding for the Study:**

This program received no specific grant from funding agencies in the public, commercial, or not-for-profit sectors.